# Complicated Hystero-Myomectomy

**Published:** 1901-06

**Authors:** Chas. Banta

**Affiliations:** Interne.


### II.—COMPLICATED HYSTERO-MYOMECTOMY.

Reported by CHAS. BANTA, M. D , Interne.

Mrs. G., aged 44, admitted to hospital April 29, 1901. No children; age at first menstruation, 13; married; abortion, one twenty years ago. Two years ago patient noticed a swelling in pelvic region, which has been gradually increasing, causing a feeling of heaviness often accompanied by dragging pains, which confined her to her bed. Has had two attacks of peritonitis. Floods profusely at menstrual period, has vertigo and dizziness. Urine diminished in quantity and of high specific gravity. Patient prepared for operation, and abdomen opened through the median line—fibroid, subperitoneal, involving the body of uterus, and growing a little posterior to fundus. The ovary on the right side three times its normal size, attached to which were a number of cysts, the size of English walnut and connected with the ovarian tissue, which was thick and cirrhotic. There was hydrosalpinx of left tube; ovary and tube lying posterior, and bound down to tumor; the sigmoid flexure was adherent, also omentum, showing evidence of severe inflammatory action. Tumor freed from its attachments by ligating either ovarian artery, separating adhesions, when the uterine wall was next treated in same manner, using No. 2, catgut. Cervix left, which was closed and covered with peritoneum. Size of mass, 7 x 5 x 4½ inches; weight, 5 lbs. Wound in abdomen closed with silkworm-gut. Stitches removed on eleventh day; union complete.

